# Editorial: Sex and Gene-Dependent Neurotoxicity

**DOI:** 10.3389/fgene.2019.00165

**Published:** 2019-03-05

**Authors:** Joanna A. Ruszkiewicz, Michael Aschner

**Affiliations:** Department of Molecular Pharmacology, Albert Einstein College of Medicine, Bronx, NY, United States

**Keywords:** sex, gene, neurotoxicity, toxicogenomic, neurotoxicology

A personalized approach is an emerging concept in neurotoxicology, which takes into consideration individual differences in susceptibility, stemming predominantly from genetic predispositions. A growing number of studies suggests that genetic factors contribute to neurological diseases (Foo et al., 2012; Little et al., 2017), and there is little doubt that this approach could benefit the neurotoxicology discipline, where heterogeneity in response to neurotoxicants occurs frequently. Genetic background plays a prominent role in the susceptibility of individuals or populations to neurotoxic agents (Llop et al., 2015) and sex is an important carrier of genetic differences inside populations. Sex widely influences brain function (McEwen and Milner, 2017). This was recently recognized by the National Institutes of Health (NIH), which now mandates the inclusion of sex as a biological variable in research projects (Clayton and Collins, 2014). Discovering the genetic- and sex-biasing factors of susceptibility to neurotoxins, not only helps to identify those individuals at higher risk for disease, but also expands upon our understanding of the mechanisms by which toxicants impair neurological function in the population at large.

The contributions to this Research Topic include reviews and original research articles, which explore different approaches in the field of neurotoxicology, highlighting genetic-, and by inference, sex-dependent susceptibility. A review by Torres-Rojas and Jones summarizes and discusses sex contribution to susceptibility to numerous toxicants, and related biological factors that influence neurotoxicity. The article presents evidence for a sex-dependent difference in response to heavy metals, ethanol, drugs of abuse, air pollutants, pesticides, and hormone disruptors, in both humans and rodents. Developmental neurotoxicity of heavy metals is a rapidly growing field of research, and its relevance to human health is also reflected in this collection. A review by Singh et al. focuses on the developmental neurotoxicity of lead, discussing sex-dependent differences in response to prenatal and postnatal lead (Pb) exposures in humans and animal models, and explores potential molecular mechanisms that may contribute to sex-dependent differences in disease outcomes. An original research paper by Steadman Tyler et al. explores sex-dependent differences in the neurodevelopmental effects of arsenic (As) exposure. The work describes sex-dependent differences in cognitive flexibility in a reversal-learning task, as well as cortical expression of histone deacetylase and brain-derived neurotrophic factor upon As exposure in mice. Moreover, the study provides evidence on the protective effect of the histone deacetylase inhibitor—valproic acid—in As-induced neurotoxicity. An article by Wahlberg et al. presents a complex, cross-sectional analysis of polymorphisms in manganese (Mn) transporters and its relevance to neurodevelopmental processes in children. The study suggests that common polymorphisms in *SLC39A8* and *SLC30A10* influence neurodevelopmental outcomes in children secondary to dysregulation of Mn levels. Human chronic exposure to mercury (Hg) remains a major public health concern, especially exposure to methylmercury (MeHg), due to fish consumption. Arrifano et al. investigated the genotypic and allelic profiles of apolipoprotein E gene (*APOE*) in Amazonian riverine populations chronically exposed to Hg. The study suggests allele-dependent Hg accumulation, with *APOE4*-carriers exhibiting the highest Hg levels.

The articles in this mini collection impart novel and timely information on sex- and gene-dependent neurotoxicity. As the field is moving forward at an accelerated pace, we hope and anticipate this collection will foster new awareness and additional studies in this domain. Albeit the content of this issue refers mostly to the neurotoxic effects of metals, we hope that highlighting this subject will facilitate recognizing sex- and gene- dependent outcomes in the future research of organic neurotoxins. We wish to thank the authors and reviewers for contributing to this Research Topic and hope the collection will stimulate further discoveries in this field.

## Author Contributions

All authors listed have made a substantial, direct and intellectual contribution to the work, and approved it for publication.

### Conflict of Interest Statement

The authors declare that the research was conducted in the absence of any commercial or financial relationships that could be construed as a potential conflict of interest.
